# Impact of Pressurized Liquid Extraction and pH on Protein Yield, Changes in Molecular Size Distribution and Antioxidant Compounds Recovery from Spirulina

**DOI:** 10.3390/foods10092153

**Published:** 2021-09-12

**Authors:** Jianjun Zhou, Min Wang, Celia Carrillo, Zhenzhou Zhu, Mladen Brncic, Houda Berrada, Francisco J. Barba

**Affiliations:** 1 Nutrition and Food Science Area, Preventive Medicine and Public Health, Food Science, Toxicology and Forensic Medicine Department, Faculty of Pharmacy, Universitat de València, Avda. Vicent Andrés Estellés, s/n, 46100 Burjassot, València, Spain; jianz@alumni.uv.es (J.Z.); minwang@alumni.uv.es (M.W.); houda.berrada@uv.es (H.B.); 2 Nutrición y Bromatología, Facultad de Ciencias, Universidad de Burgos, 09001 Burgos, Spain; 3 College of Food Science and Engineering, Wuhan Polytechnic University, Wuhan 430023, China; zhenzhouzhu@126.com; 4 Faculty of Food Technology and Biotechnology, University of Zagreb, Pierottijeva 6, 10000 Zagreb, Croatia; mladen.brncic@pbf.unizg.hr; 5 Nutrition and Bromatology Group, Department of Analytical and Food Chemistry, Faculty of Food Science and Technology, Ourense Campus, University of Vigo, 32004 Ourense, Spain

**Keywords:** microalgae, PLE, RSM-CCD, SDS-PAGE, Triple TOF–LC–MS–MS, bioactive compounds

## Abstract

The research aims to extract nutrients and bioactive compounds from spirulina using a non-toxic, environmentally friendly and efficient method—Pressurized Liquid Extraction (PLE). In this work, Response Surface Methodology (RSM)–Central Composite Design (CCD) was used to evaluate and optimize the extraction time (5–15 min), temperature (20–60 °C) and pH (4–10) during PLE extraction (103.4 bars). The multi-factor optimization results of the RSM-CCD showed that under the pressure of 103.4 bars, the optimal conditions to recover the highest content of bioactive compounds were 10 min, 40 °C and pH 4. Furthermore, the compounds and antioxidant capacity of PLE and non-pressurized extraction extracts were compared. The results showed that under the optimal extraction conditions (10 min, 40 °C and pH 4), PLE significantly improved the antioxidant capacity (2870.5 ± 153.6 µM TE), protein yield (46.8 ± 3.1%), chlorophyll a (1.46 ± 0.04 mg/g), carotenoids (0.12 ± 0.01 mg/g), total polyphenols (11.49 ± 0.04 mg/g) and carbohydrates content (78.42 ± 1.40 mg/g) of the extracts compared with non-pressurized extraction (*p* < 0.05). The protein molecular distribution of the extracts was analyzed by sodium dodecyl sulfate polyacrylamide gel electrophoresis (SDS-PAGE), and the results showed that there were more small-molecule proteins in PLE extracts. Moreover, Liquid Chromatography Triple Time of Flight Mass Spectrometry (TOF–LC–MS–MS) was used to analyze the phenolic profile of the extracts, and the results showed the extracts were rich on phenolic compounds, such as p-coumaric acid and cinnamic acid being the predominant phenolic compounds in the PLE extract. This indicates that PLE can promote the extraction of bioactive compounds from Spirulina, which is of great significance for the application of PLE technology to obtain active substances from marine algae resources.

## 1. Introduction

Functional foods are generally foods rich in ingredients that can provide or promote specific beneficial effects for human health. These ingredients, such as proteins, lipids, vitamins and trace elements, can be derived from microalgae [1,2]. There are more than 1000 kinds of algae in the ocean, and they are used as an important food source in many countries, such as China, Japan and South Korea. Microalgae have been also introduced into Western countries as a natural food rich in nutrients [3,4].

Among the numerous species of algae, spirulina (*Arthrospira platensis*), a cyanobacteria, has been extensively studied due to its commercial importance as a source of proteins, vitamins and fatty acids [5]. Moreover, according to recent reports, spirulina has the highest protein content (70% on a dry basis) among algae organisms and contains a large amount of essential amino acids [6,7]. At present, spirulina is used worldwide as a source to extract biological compounds, mainly protein, pigments and compounds with antioxidant properties [8]. Therefore, spirulina has the potential to provide solutions for the food, pharmaceutical and cosmetic industries. However, many processes are required before microalgae can be used in these industries, involving microalgae cultivation, functional ingredients recovery and safety evaluation of the ingredients recovered [9]. At present, the cultivation technology of microalgae has matured. Therefore, the main problem in practical industrial applications is how to use green, highly efficient and low-cost extraction methods to make the bioactive compounds of spirulina useful in the industry.

Traditional extraction methods include dipping (soaking), percolation, countercurrent extraction and Soxhlet extraction [10,11]. However, the rigid cell wall structure of spirulina is disadvantageous for traditional extraction methods [12]. Traditional methods increase the extraction temperature (over 100 °C) and volume of organic reagents (chloroform, ether, etc.) and extend the extraction time to achieve the purpose of obtaining algae biomass [13]. Although heating and organic solvents increase the yield of the target product, it has a negative impact on the substances obtained, such as destroying the structure and function of some compounds [14]. Therefore, a growing interest regarding the use of low-temperature, non-toxic and efficient extraction techniques has been shown.

Compared with traditional extraction technology, the Pressure Fluid Extraction (PLE) method has advantages in protecting the functional ingredients and shortening the extraction time [15,16]. PLE is a fully automatic extraction technology that uses a combination of elevated temperature and pressure to obtain bioactive compounds in a short time. In addition, PLE can process multiple samples in the same batch with a low reagent usage [17]. PLE has been used in the fields of food and medicine and achieved good results [18,19]. In this respect, a study used Irish seaweed *Ascophyllum nodosum*, *Fucus vesiculosus* and *Fucus serratus* as the extraction raw materials, 80% ethanol aqueous solution as the extraction reagent and pressure solvent extraction (1000 psi) as the extraction method and obtained extracts with high antioxidant capacities, based on their ability to protect against oxidant-induced DNA damage [20]. Similarly, Denery et al. used pressurized extraction solvent technology (1500 psi, 40 °C, 10 min) to extract astaxanthin from *Haematococcus pluvialis* and reached up to 9.5 mg/g and 8.4 mg/g of astaxanthin recovered with acetone and ethanol, respectively [21].

However, to the best of our knowledge, there are few reports using PLE to obtain bioactive compounds from spirulina. Research at this respect is mainly focused on ultrasound, microwave assisted extraction and pulsed electric fields [12,22,23,24]. Therefore, using PLE as an extraction technology to obtain active substances from spirulina and optimizing the extraction process to get as many functional ingredients as possible have important economic value and significance for the utilization of spirulina resources.

In this study, PLE was used to extract spirulina nutrients and bioactive compounds using deionized water at different pH (4–10), mild temperatures (20–60 °C) and short extraction times (5–15 min) with a constant pressure of 103.4 bars. Further, the compounds (protein, carbohydrate, chlorophyll a, chlorophyll b, carotenoids and polyphenol) and the antioxidant properties of the extracts obtained were analyzed.

## 2. Materials and Methods

### 2.1. Chemical and Reagents

AAPH (2,2′-Azobis(2-methylpropionamidine) dihydrochloride), Folin–Ciocalteu reagent, gallic acid, Trolox (6-hydroxy-2,5,7,8-tetramethylchroman-2-carboxylic acid), D-glucose, phenol reagent, fluorescein sodium salt and potassium persulfate (K_2_S_2_O_8_) were purchased from Sigma–Aldrich (Steinheim, Baden-Württemberg, Germany). Sodium carbonate (Na_2_CO_3_) was acquired from VWR (Saint-Prix, France). SDS (sodium dodecyl sulfate, purissimum-CODEX) was obtained from Panreac (Barcelona, Spain), sodium hydroxide, glacial acetic acid and sulfuric acid were supplied by Fisher Scientific (Madrid, Spain). Acetonitrile (HPLC grade), acetone, glycerol and bromophenol blue indicator (ACS reagent) were supplied by Merck (Darmstadt, Germany). The Silica (SiO_2_) was purchased from Supelco Analytical™, diatomaceous earth and other materials for generation of PLE extracts were purchased from Dionex (Dionex, Leeds, UK).

### 2.2. Samples

Spirulina biomass comes from *Arthrospira platensis* species, strain paracas 15016, Paracas being the lake from where it originally comes (Lima, Peru). Cultivation took place at EcoSpirulina company (Serra, Valencia, Spain) in raceway ponds using a greenhouse under natural sunlight without any artificial light added. Shadow nets partially covered the cultivation ponds, thus allowing to control phytopigments production. At the time of the experiment, day-time temperature was 32 °C on average, while temperature decreased to 24 °C at night. Culture’s pH varied between 9.8 and 10.4. It was regulated by the addition of CO_2_ at the time of harvesting, which took place daily. Then, biomass was filtered using a tambor filter of 31 micrometers at 6–10 rpm. Cultivation medium went back to the cultivation pond, while biomass was vacuum-pressed and then frozen in 50 g portions. Frozen biomass was used to carry out the experiments at the laboratory of Nutrition and Food Science, Faculty of Pharmacy, Universitat de València.

### 2.3. PLE Extraction Process

Spirulina samples had a moisture content of 74.2% ± 2.1% (*w*/*w*). After freeze-drying, they were transferred into a grinder for pulverization for subsequent experiments. Before PLE extraction, spirulina was pretreated according to the experimental method previously reported by O’Sullivan et al. [20]. Five hundred milligrams of spirulina sample were mixed with 1.5 g silica in a ratio of 1:3 (*w*/*w*) and 1.0 g diatomaceous earth in a ratio of 1:2 (*w*/*w*). After mixing well with a mortar, all the mixture was transferred into the PLE extraction cell. Before the extraction, an initial heating up of the cell was performed, and the cell was rinsed by the extractant reagent. The extraction reagent was purged from the cell under a nitrogen pressure of 103.4 bars. Cleaning procedures were used between different groups to prevent residual extracts. After the extraction procedure was completed, the sample in the collection bottle was transferred to 15 mL centrifuge tubes. The sample was stored at −20 °C for subsequent experimental analysis.

### 2.4. Total Protein Determination 

The total protein content in the sample was tested by Kjeldahl method. Briefly, 2 mL of spirulina extract, 3 g of potassium thiosulfate, 6 zeolites, 5 drops of copper sulfate and 5 mL of concentrated sulfuric acid were added to the nitrification tube. Then, it was heated at 120 °C until the color was clear and bright. Automatic Kjeldahl nitrogen analyzer was used to collect ammonia. Finally, the distillate was collected in a boric acid solution (2.0% *w*/*v*) and titrated using a hydrochloric acid solution (0.014 mol/L), methyl orange was used as the end point indicator of the titration. The formula previously described by de Lourdes Mendes Finete et al. was used for calculating the nitrogen contents [25], using the conversion factor of protein content according to Grossmann et al. [26]. The calculation formulae of nitrogen and protein content are as follows:

(1) $$\mathrm{Nitrogen}\;\%\;=\frac{\left[\left(\mathrm{mL}\;\mathrm{Standard}\;\mathrm{acid}-\mathrm{mL}\;\mathrm{blank}\right)\times N\;\mathrm{of}\;\mathrm{acid}\times 1.400\right]}{\left(\mathrm{Weight}\;\mathrm{of}\;\mathrm{sample}\;\mathrm{in}\;\mathrm{grams}\right)}$$

(2) Protein %=6.5×Nitrogen % 

### 2.5. Total Carbohydrate Content Determination

The carbohydrate content was tested by the concentrated sulfuric acid-phenol method [27]. Specifically, 1 mL of sample, 0.5 mL of phenol and 2.5 mL of concentrated sulfuric acid were sequentially added to a glass reaction flask and reacted for 30 min at room temperature, and then, the absorbance value at 490 nm was measured by spectrophotometry. D-glucose was used as a standard for calculating carbohydrate content.

### 2.6. Pigments Determination 

The determination of the pigments (chlorophyll a, chlorophyll b and carotenoids) was carried out according to the method previously described by Parniakov et al. [12]. After the sample was thawed, it was centrifuged at 4 °C, 18,407× *g* for 5 min, and the absorbance value was measured at the wavelengths of 665, 652 and 470 nm, respectively. The calculation of chlorophyll a, chlorophyll b and carotenoids contents were according to the equations previously described by Wellburn et al. [28].

(3) $$C_{\mathrm{ch}}^{a}=16.82A_{\mathrm{ch}}^{a}-9.28A_{\mathrm{ch}}^{b}$$

(4) $$C_{\mathrm{ch}}^{b}=36.92A_{\mathrm{ch}}^{b}-16.54A_{\mathrm{ch}}^{a}$$

(5) $$C_{\mathrm{cr}}=\frac{\left(1000A_{\mathrm{cr}}-1.91C_{\mathrm{ch}}^{a}-95.15C_{\mathrm{ch}}^{b}\right)}{225}$$

where $C_{\mathrm{ch}}^{a}$, $C_{\mathrm{ch}}^{b}$ and $C_{\mathrm{cr}}$ are the concentrations of chlorophyll a, chlorophyll b and total carotenoids, respectively.

### 2.7. Polyphenols Determination

The total polyphenol content in the sample was analyzed using the Folin–Ciocalteu method [12]. Specifically, 0.2 mL of spirulina extract, 1 mL of Folin–Ciocalteu solution (dilution with water, 1:10, *v*/*v*) and 0.8 mL sodium carbonate solution (75 g/L) were mixed and then reacted for 10 min in a water bath at 50 °C under darkness. Finally, the absorbance value of the reaction solution at 750 nm was measured by spectrophotometry. Gallic acid was used to calibrate the total polyphenol content in the sample. 

The identification and quantification of the major phenolic compounds present in the spirulina extracts was carried out on TripleTOF™ 5600 (ABSCIEX) LC/MS/MS system equipped with Agilent 1260 Infinity (Agilent, Waldbronn, Germany). Chromatographic separation was performed on a Waters UPLC C18 column 1.7 µm (2.1 × 50 mm) Acquity UPLC BEH.C18 from Waters (Cerdanyola del Vallès, Spain). The mobile phase was composed of water (0.1% formic acid, A) and methanol (0.1% formic acid, B). The gradient elution of the mobile phase was as follows: from 0 to 13 min, 90% (A) and 10% (B); from 13–15 min 100% (B); from 15.1–22 min, 90% (A) and 10% (B). The flow rate and injection volume were 0.4 mL/min and 5 μL, respectively. The MS acquisition was under a mass range of 80–1200 m/z. The calibration was performed applying an external calibration delivery system, which infuses the calibrating solution before sample introduction. The MS was operated using an Information Dependent Acquisition (IDA) method with the survey scan type (TOF-MS) and the dependent scan type (Product Ion) at −50 V of collision energy. The MS parameters were as follows: ion spray voltage of −4500 V, declustering potential of 90 V, collision energy of −50 V, temperature at 400 °C with curtain gas of 25 psi, ion source gas 1 at 50 psi and ion source gas 2 at 50 psi. IDA MS/MS was performed using the following criteria: ions that exceeded 100 CPS, ion tolerance 50 MDa, collision energy fixed at 25 V and dynamic background subtraction activated. The quantification was based on the use of standards of the polyphenols corresponding to the main groups of compounds identified.

### 2.8. Antioxidant Capacity

The Oxygen radical absorbance capacity (ORAC) was used to determine the antioxidant capacity of the extract. The method previously described by Du et al. with slight modifications was used [29]. AAPH was used as a peroxide generator and Trolox as an antioxidant standard. Each sample was added to 5 wells in parallel and repeated 3 times. The microplate reader was programmed to record the fluorescein fluorescence of each cycle. Kinetic readings were recorded as 45 cycles, and each cycle was set to 60 s. PBS (75 mM, pH 7.4) was used as a blank and to configure Trolox standards. Briefly, 50 μL of extract and 50 μL of fluorescein solution were added to the 96-well plates and then incubated in a microplate reader for 30 min; then, 25 μL peroxide AAPH was added to the 96-well plates to initiate the oxidation reaction. To calculate the ORAC value, the linear equation between the Trolox standard or sample and the net area under the fluorescence decay curve was used. The calculation formula was as follows:

(6) $$\mathrm{ORAC}\left(\mathrm{Trolox}\right)=\frac{\left(A_{\mathrm{Sample}}-A_{\mathrm{Blank}}\right)}{\left(A_{\mathrm{Trolox}}-A_{\mathrm{Blank}}\right)}$$

Trolox equivalent antioxidant capacity (TEAC) referred to related literature and slightly modified was used to evaluate the antioxidant capacity of the extract [30]. Briefly, mix 25 mL of 7 mM 2,2′-azidobis-(3-ethylbenzothiazolin-6-sulfonate) (ABTS) with 440 µL of 140 mM potassium thiosulfate solution and store the mixed solution in the dark at room temperature for 12 to 16 h as working solution. Dilute the working solution with 96% ethanol to keep the absorbance value between 0.700 ± 0.020. During the test, mix 0.1 mL sample or standard solution with 2 mL working solution, and after reacting for 3 min in a dark room, read the absorbance value of the reaction solution at 734 nm. Trolox at different concentrations was used as the standard product for antioxidant capacity calibration.

### 2.9. Protein Molecular Size Distribution (SDS-PAGE Gels)

To evaluate the protein molecular size distribution, the method described by Jong et al. was used with slight modifications [31]. Firstly, the buffer was prepared. For this purpose, 0.5 g of SDS, 2.46 g of Tris-HCl, 6.25 mL of glycerol, 2.5 mL of bromophenol blue reagent and 13 mL of water were combined in the beaker and mixed well using a magnetic stirrer under darkness conditions. Then, 8 mg of Dithiothreitol (DTT) was added to 500 μL of the sample buffer, named as ‘A’. Fixed working solution was configured as follows, 400 mL methanol + 100 mL acetic acid + 500 mL water. The decolorizing solution was 200 mL methanol + 100 mL acetone + 700 mL deionized water. Moreover, to prepare the electrophoresis solution, 0.5 g SDS, 7.2 g Glycine and 1.515 g Trizma base were dissolved in 1000 mL distilled water. Then, 100 μL of Spirulina extract was taken and mixed with 400 μL of acetone to prepare the gels. Then, the mixture was vortexed for 10 s and centrifuged at 11,000 rpm and 4 °C for 10 min. The supernatant was removed and put it in a fume hood for 5 min to volatilize the remaining acetone reagent. Then, 100 μL of water was added to rinse the precipitate, and the mixture was immersed in an ultrasound bath at 25 °C for 20 s to fully dissolve the precipitate, named as ‘B’. Then, ‘A’ and ‘B’ were mixed (20:20, *v*/*v*) and heated at 95 °C for 5 min. The Bio-Rad device for gel electrophoresis was used, and the Marker and sample loading volumes were 10 μL and 25 μL, respectively. A constant voltage of 80 V was used, and the electrophoresis was finished when the marker band reached the bottom of the gel. After soaking the gel in the fixative for 30 min, the Coomassie Brilliant Blue dye solution was added and kept for 30 min. Finally, the decolorizing solution was added and kept shaking for 24 h until the protein bands in the gel were clear. Image-J was used to calculate the area of the bands. 

### 2.10. Experimental Design and Statistical Analysis

Three factors (temperature, time, pH) and three levels of the response surface full factorial design experiment were used to optimize the PLE extraction. Two central points were selected to establish the experimental error, and a total of 16 experiments were carried out with a random sequence. The experimental variables were the following: temperature, 20–60 °C; time, 5–15 min and pH, 4–10. There are 7 response factors, followed by content of protein, carbohydrates, chlorophyll a, chlorophyll b, carotenoids, polyphenol and ORAC. The 16 experiments are shown in Table 1.

All results are presented as mean ± standard deviation. SPSS was used to analyze the data for variance analysis, and Duncan’s test was performed; *p* < 0.05 indicates that the data are significantly different, *p* < 0.01 indicates that a very significant difference exists. Image-J software was used for the electrophoresis band analysis, selecting 8-bits as the image type and a gray level of 50%. 

## 3. Results and Discussion

### 3.1. Effect of PLE on High-Added-Value Compounds

In this study, the protein extraction rate, carbohydrate content and antioxidant properties of the extracted samples were analyzed. Table 2 shows the compound content in the 16 extracts under different conditions respectively.

The results show that the protein extraction rate ranged from 14% to 48%, observing higher protein yield at 40 °C, 10 min and pH 4 (47.87%) and 40 °C, 10 min and pH 10 (45.81%). This is significantly higher than the extraction rate obtained after applying PLE at 20 °C, 15 min and pH 4 (14.02%) and 20 °C, 5 min and pH 10 (14.43%) (*p* < 0.05). From the results obtained, it can be observed that 40 °C seems to be the better temperature to promote protein extraction compared to 20 °C. Moreover, from the obtained regression Equation (7), higher temperatures and longer extraction times provided a higher protein recovery, while a negative effect regarding protein extraction was found when the pH was high. 

**Figure 1:**
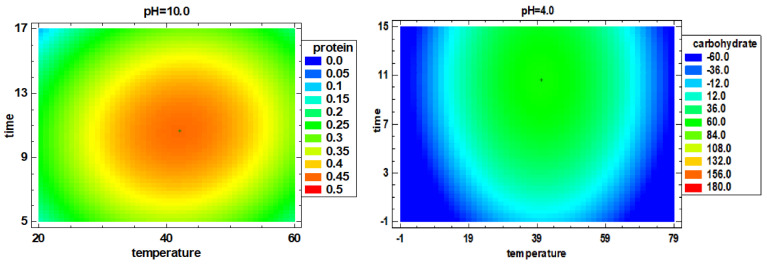
Estimated response surface of protein extract efficiency (%) and carbohydrate content (mg/g).

The model F-value of 5.84 (*p* value = 0.022) implies the model is significant. There is a 2.36% chance that the lack of Fit F-value could be due to the noise. The optimized conditions for protein extraction are shown in Figure 1, obtaining the optimal extraction conditions after PLE at 42 °C, 10.7 min, pH 10.

(7) $$\mathrm{Protein}\;\%=-4.718+3.092\times T+6.942\times t-18.646\times \mathrm{pH}-0.040\times T^{2}\;+0.019\times T\times t+0.010\times T\times \mathrm{pH}-0.360\times t^{2}-0.004\times t\times \mathrm{pH}\;\;+1.317\times {\mathrm{pH}}^{2}\left(R^{2}=0.898,\;P=0.022\right)$$

The results in Table 2 show that the carbohydrate content of the extracts obtained ranged from 6.9 mg/g to 81 mg/g. The results showed that the extraction amount of carbohydrates at 40 °C, 10 min and pH 4 and 40 °C, 10 min and pH 10 were 80.95 mg/g and 58.90 mg/g, respectively, which was significantly higher than other experiments, especially after PLE at 5 min and pH 4 either at 20 °C (6.90 mg/g) or 60 °C (8.81 mg/g) (*p* < 0.05). These results show that changing the extraction temperature and time with the same pH significantly changes the carbohydrate content of the spirulina extracts (*p* < 0.05). From the obtained regression Equation (8), similarly to protein recovery, an increased carbohydrate extract efficiency can be seen when temperature and time were increased, while a negative effect was found for pH. The model F-value of 4.45 implies that the model is significant (*p* value = 0.026). There is a 9.28% chance that a lack of fit F-value could be due to the noise. The optimal conditions are shown in Figure 1, being 40.3 °C, 10.6 min and pH 4 the optimal conditions to achieve the highest carbohydrate recovery.

(8) $$\mathrm{Carbohydrate}\frac{\mathrm{mg}}{g}=-33.734+6.862\times T+11.680\times t-36.168\times \mathrm{pH}-0.084\times T^{2}\;\;-0.541\times t^{2}-0.023\times t\times \mathrm{pH}\;+2.601\times {\mathrm{pH}}^{2}\;\left({\mathrm{Adjusted}\;R}^{2}=0.617,\;P=0.026\right)\;$$

Moreover, the contents of chlorophyll a, chlorophyll b and carotenoids in the extracts obtained were analyzed. For chlorophyll a, the extract content at 40 °C, 10 min and pH 4 (1.406 mg/g) was significantly (*p* < 0.05) higher than in the other experiments. For chlorophyll b, relatively high extraction amounts were found at 40 °C, 10 min and pH 4 (2.017 mg/g) and 40 °C, 10 min and pH 10 (1.626 mg/g) (*p* < 0.05). Likewise, the carotenoid content was lower than that observed for chlorophyll a and chlorophyll b, although similarly to chlorophyll a and chlorophyll b, the highest extraction rate for carotenoids (0.648 mg/g) was found at 40 °C, 10 min and pH 4. 

**Figure 2:**
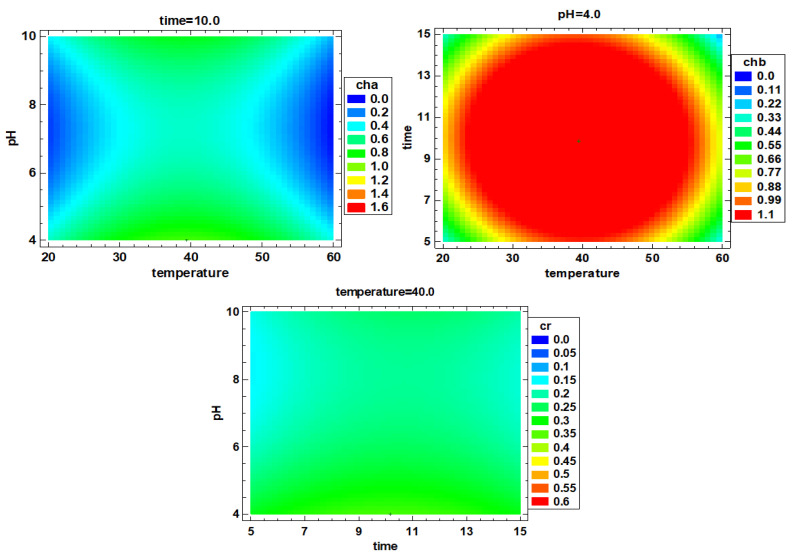
Estimated response surface of chlorophyll a (mg/g), chlorophyll b (mg/g) and carotenoids (mg/g). cha, chb and cr correspond to chlorophyll a, chlorophyll b and carotenoids, respectively.

From the regression Equations (9)–(11), a positive effect of temperature and time regarding chlorophyll a, chlorophyll b and carotenoid extraction could be observed, while pH had a negative effect. The model F value for chlorophyll b is significant (F-value = 4.19; *p*-value = 0.027). However, the model F-values for chlorophyll a and carotenoids are not significant (*p* value > 0.05), indicating that these models cannot well summarize the extraction scheme of chlorophyll a and carotenoids. In this experiment, an aqueous solution was used as the extraction reagent, so the content of chlorophyll a/carotenoids (fat-soluble pigments) was low; then, these equations can be used as a reference, and Figure 2 shows the optimized extraction conditions for chlorophyll a, chlorophyll b and carotenoids were 39.7 °C, 10.0 min and pH 4; 39.4 °C, 9.4 min and pH 4 and 39.6 °C, 10.3 min and pH 4, respectively. From the above results, it could be seen that the extraction conditions of the three pigments are similar, that is, almost at 40 °C, 10 min and pH 4.

(9) $$\mathrm{Chlorophyll}\;a\frac{\mathrm{mg}}{g}=-0.251+0.088\times T+0.206\times t-0.564\times \mathrm{pH}-0.001\times T^{2}\;-0.010\times t^{2}+0.039\times {\mathrm{pH}}^{2}\;\left(\mathrm{Adjusted}\;R^{2}=0.608,\;P=0.131\right)$$

(10) $$\mathrm{Chlorophyll}\;b\frac{\mathrm{mg}}{g}=-0.503+0.156\times T+0.438\times t-1.109\times \mathrm{pH}-0.002\times T^{2}\;-0.022\times t^{2}+0.078\times {\mathrm{pH}}^{2}\;\left(\mathrm{Adjusted}\;R^{2}=0.560,\;P=0.027\right)$$

(11) $$\;\mathrm{Carotenoids}\;\mathrm{mg}/g=-0.279+0.039\times T+0.048\times t-0.134\times \mathrm{pH}\;-0.002\times t^{2}\;+0.001\times t\times \mathrm{pH}+0.008\times {\mathrm{pH}}^{2}\;\left(\mathrm{Adjusted}\;R^{2}=0.475,\;P=0.075\right)$$

**Figure 3:**
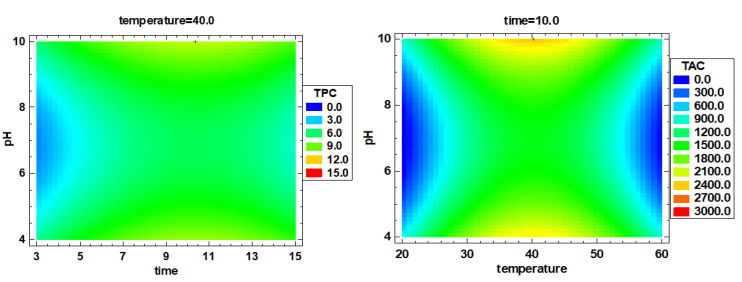
Estimated response surface of total polyphenol content and total antioxidant capacity. TPC (mg/g) and TAC corresponding to total polyphenol content and total antioxidant capacity (µM TE).

Regarding total polyphenols extraction, the optimal recovery was achieved at 40 °C, 10 min and pH 4 and 40 °C, 10 min and pH 10, corresponding to 11.61 mg/g and 10.85 mg/g, respectively. However, when the temperature was 20 °C and the pH = 4, even if the time was increased from 5 min to 15 min, the yield of polyphenols was still very low, corresponding to 0.97 mg/g and 0.63 mg/g, respectively. This indicates that for the extraction content of polyphenols, 40 °C is significantly better than 20 °C. Equation (12) shows the positive effect of increased temperatures and longer extraction times and the negative effect of increased pH. The model F-value of 4.63 implies the model is significant (*p* value = 0.029). The optimized conditions obtained from response surface plot for polyphenol are 39.9 °C, 10.4 min and pH 10 (Figure 3).

(12) $$\mathrm{Polyphenol}\frac{\mathrm{mg}}{g}=-6.604+1.169\times T+1.570\times t-5.570\times \mathrm{pH}-0.014\times T^{2}\;+0.006\times T\times \mathrm{pH}-0.080\times t^{2}+0.424\times {\mathrm{pH}}^{2}\;(\mathrm{Adjusted}\;R^{2}=0.659,\;P=0.029)\;$$

Oxygen free radical scavenging capacity was used to evaluate the total antioxidant capacity of 16 samples. Table 2 shows that the antioxidant value of the samples varies greatly, ranging from 100 to 3000, which mainly depends on the biomass content (protein, polyphenols, carbohydrates and pigments) analyzed previously.

The samples extracted under the conditions of 40 °C, 10 min and pH 4 had the highest antioxidant value of 2915 µM TE, which is similar to the sample obtained under 40 °C, 10 min and pH 10, which had 2831 µM TE. There was no significant difference in the antioxidant value of the two samples. It is worth noting that when the extraction conditions were 40 °C, 10 min and pH 7 and 40 °C, 15 min and pH 7, the antioxidant capacity of the sample was relatively strong, the values being 1491.0 µM TE and 1595.4 µM TE, respectively. The antioxidant value of the above four groups is much higher than other groups (between 100–500 µM TE). It can be inferred that temperature has an important influence on the antioxidant value of the extract, especially at 40 °C.

(13) $$\mathrm{ORAC}\;µM\;\mathrm{TE}=-0.279+0.039\times T+0.048\times t-0.134\times \mathrm{pH}\;\;-0.002\times t^{2}+0.001\times t\times \mathrm{pH}\;+0.008\times {\mathrm{pH}}^{2}\;\left(\mathrm{Adjusted}\;R^{2}=0.687,\;P=0.053\right)$$

Equation (13) shows a positive effect of temperature and time on ORAC values, whereas pH had a negative effect. The model F-value of 3.30 implies that the model is significant (*p* value = 0.053). The optimized conditions for ORAC are 40.2 °C, 10.2 min and pH 10 (Figure 3). 

### 3.2. Multiple Factor Response Results and Verification Experiments

**Figure 4:**
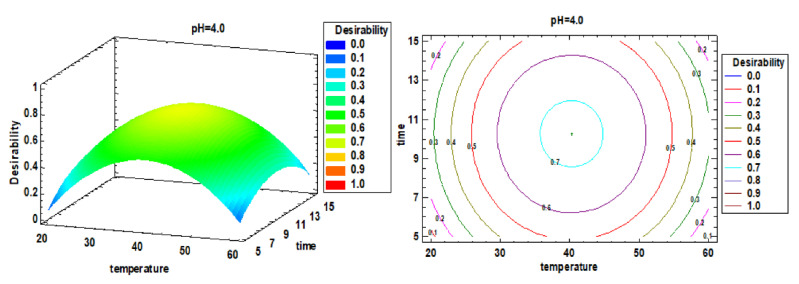
Estimated response surface and contour plots for multiple factor responses.

According to the single-factor optimization, the multi-factor optimization analysis was performed, and the response surface diagram is shown in Figure 4. According to the comprehensive optimization results of all extract indexes, the optimal extraction conditions were determined to be 40.3 °C, 10.3 min and pH 4. The RSM-CCD predicted value is compared with the verified experimental value (predicted value vs. verified value), that is, the protein extraction rate was 43.5 ± 1.9% vs. 46.8 ± 3.1%, chlorophyll a was 0.89 ± 0.13 vs. 1.46 ± 0.04 mg/g, chlorophyll b was 1.86 ± 0.13 vs. 2.06 ± 0.15 mg/g, carotenoids were 0.12 ± 0.01 vs. 0.14 ± 0.02 mg/g, the TPC was 9.64 ± 1.75 vs. 11.49 ± 0.04 mg/g, the carbohydrate content was 69.3 ± 1.8 vs. 78.4 ± 1.4 mg/g and the ORAC value was 2297 ± 338.5 vs. 2870.5 ± 153.6 µM TE (*p* > 0.05). There were no significant differences of the results between the verification experiment and group 9 (*p* > 0.05). Therefore, using deionized water as extraction reagent, 40 °C, 10 min and pH 4 provides the best extraction conditions. 

### 3.3. Effect of Pressure on Compounds Extraction and Antioxidant Capacity

RSM-CCD optimization experiments showed a significant impact of temperature, time, and pH on the extraction of spirulina active substances (*p* < 0.05). Throughout the extraction process, nitrogen pressure was constant at 103.4 bars. It is worth discussing whether the constant pressure of 103.4 bars during the PLE extraction process played a key role in the extraction of spirulina active substances. Therefore, a control experiment focused on the effect of pressure on the extraction of bioactive substances in spirulina was carried out. Specifically, spirulina powder and pH 4 deionized water were mixed in proportions (0.5 g/40 mL), and samples without pressure extraction were obtained under the conditions optimized by RSM-CCD at 40 °C and 10 min. Then, the protein, chlorophyll a, chlorophyll b, carotenoids, polyphenols, carbohydrates content and antioxidant capacity (ORAC and ABTS) of the control samples were analyzed. 

**Figure 5:**
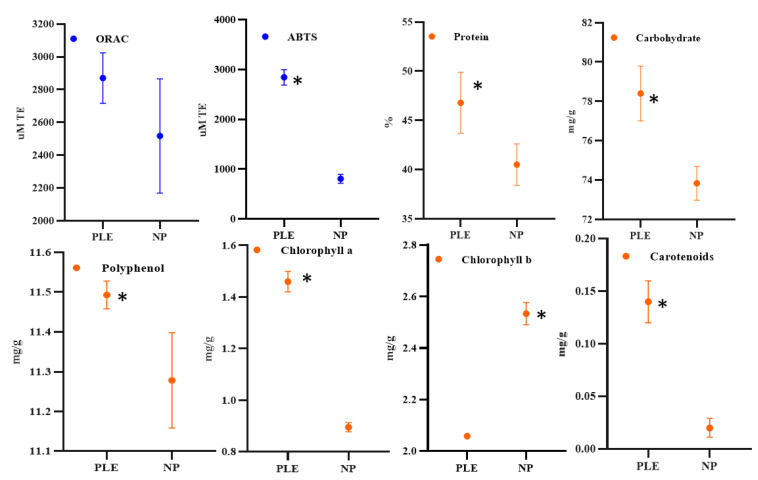
Biomass and antioxidant capacity of PLE and non-pressurized (NP) extracts. Note: * indicate significant differences (*p* < 0.05).

As it is shown in Figure 5, no significant differences were observed for ORAC results when the PLE sample and the non-pressurized one when compared, the results ranging from 2500 to 2800 µM TE. On the other hand, the ABTS results showed that the antioxidant capacity of the PLE extracts was significantly higher than the control group (*p* < 0.05), and the results were close to 2300 µM TE and 800 µM TE, respectively. Obviously, ORAC and ABTS showed a different behavior, which is attributed to the different mechanism of action. Both ORAC and ABTS can test the antioxidant capacity of the sample. The former is to examine the ability of the sample to scavenge ROO, OH and other free radicals, and the latter is to examine the ability of the sample to scavenge ABTS+ free radicals. Since the type of free radicals removed by the samples are different, the results are also inconsistent [32], although differences between the two assays, such as the corresponding end point times, could also explain the results observed. Spirulina has been extensively studied due to its great content of various functional compounds. A previous study used accelerated solvent extraction combined with different solvents (hexane, petroleum ether, ethanol and water) to evaluate the extraction of antioxidant compounds from spirulina [23]. The authors investigated the effects of different temperatures (60, 115 and 170 °C) and times (3, 9 and 15 min) on the antioxidant capacity of the extract. The results showed that using ethanol as the extraction reagent, samples with higher antioxidant capacity can be obtained at 170 °C and 15 min. Compared with our study, the extraction temperature selected in that study is too high, which is unfavorable for obtaining heat-sensitive components such as pigments, polyphenols and proteins from spirulina. Unfortunately, their study only examined the total antioxidant activity of the extracts and did not analyze any components in the spirulina extract. Moreover, we selected water as the extractant and analyzed a variety of functional substances, which reduced the cost of the reagent (compared to ethanol) and obtained a variety of bioactive compounds. 

The protein concentration in the control sample was approximately 120 mg/g, which was significantly (*p* < 0.05) lower than the protein concentration in the PLE group (138 mg/g). PLE also increased significantly (*p* < 0.05) the carbohydrate concentration (approximately 78 mg/g vs. 74 mg/g) (*p* < 0.05) under the pressure of 103.4 bars as well as the concentration of chlorophyll a, carotenoids, and polyphenols compared to control. However, the concentration of chlorophyll b was significantly (*p* < 0.05) reduced.

Regarding the effect of pressure treatment on spirulina extract, another work explored that different pretreatment methods (autoclaving at 121 °C with 103.4 kPa for 30 min; ultrasonication using a probe sonication 20% maximum power for 60 min; high-pressure homogenization at 103.4 MPa) affect the extraction of phycocyanin from spirulina [33]. The results showed that under optimized extraction conditions (dissolution step: pH 11.38, 35 min; precipitation step: pH 4.01, 60 min), the samples processed by high pressure homogenization contained more proteins, essential amino acids and other high value unsaturated fatty acids. This is consistent with our research results, that is, pressurization promotes the extraction of spirulina bioactive substances. According to the principle of pressure extraction, the strong interaction between the solute and the matrix caused by van der Waals forces or hydrogen bonds and the dipole attraction of solute molecules and the active sites of the sample matrix can be greatly reduced under high temperature and high pressure. This speeds up the extraction process of the solute molecules, reduces the activation energy required for the analysis process and reduces the viscosity of the solvent, thereby reducing the resistance of the solvent to the sample matrix and promoting the solvent to diffuse into the sample [34]. Therefore, compared with non-pressurized extraction, the pressure of 103.4 bars in this study significantly increased the protein, carbohydrate, chlorophyll a, carotenoids and polyphenols in the spirulina extract.

In addition to the PLE extraction technique, other green, non-toxic and efficient extraction methods, such as ultrasonic and microwave-assisted extraction, have also been used to obtain biologically active compounds from spirulina. The results of a recent experiment showed that ultrasonic treatment (20 kHz) promotes the mass transfer process (efficient diffusivity) in the extraction process. Compared with traditional extraction methods, ultrasonic treatment significantly increased the extracted protein content (8.63 ± 1.15 g/100 g DW vs. 229.42 ± 1.15 g/100 g DW). Under the microscope, the perforation and fragmentation of the ultrasonic treatment samples were observed, which promoted the extraction of biologically active compounds in spirulina [35]. Another study also showed that compared to the Soxhlet extraction (80 °C and 8 h), the more efficient extraction process for extracting biomolecules from spirulina is to perform ultrasonic-assisted extraction in ethanol-chloroform-water-Na_2_SO_4_ and ethanol after 20 s of water extraction in a microwave oven. Moreover, the microwave-assisted extraction method is more efficient than the ultrasonic extraction method alone [22]. In general, PLE, ultrasound, and microwave technology all show advantages in extracting active substances from spirulina. It is worth mentioning that our research does not use organic reagents, which are cleaner and cheaper than the extraction process in the above research.

### 3.4. SDS-PAGE Analysis of Protein Molecules in Spirulina Extracts

**Figure 6:**
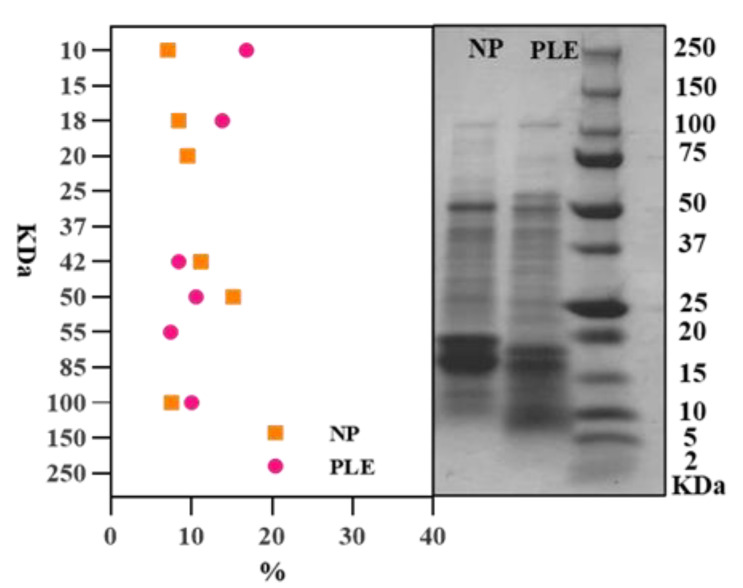
Protein molecular weight distribution of the extracts—SDS-PAGE.

Figure 6 shows the protein molecular weight distribution in spirulina extracts, ranging from 2 to 250 kDa. The protein molecular weights of the PLE extracts are distributed in 100, 75 to 50, 37, 20 to 15 and 10 kDa, the control extracts are distributed in 100, 75, 50 to 37, 20 and 10 kDa. The difference of the band distribution indicates that PLE extraction could affect the protein molecule in spirulina extracts. Image-J is used to calculate the area value of each band, and its relative proportion is shown in Figure 6. The results show that the proportion of bands between 100 to 20 kDa in the non-pressurized extracts is greater than that of the PLE-extracted group. However, when the molecular weight is lower than 20 kDa, PLE group is more than that of the non-pressurized group. This indicates that the content of small molecular weight protein in the extracted samples of the PLE group is higher than that of the non-pressurized group. There are two possible reasons for this phenomenon. One hypothesis is that more small-molecular-weight proteins are eluted due to the pressurizing effect during the extraction process of PLE. Another hypothesis is that the pressurization of PLE may affect the protein structure, resulting in more small-molecule peptides. Moreover, some previous studies have shown that pressurization can affect the structure of protein molecules in food, that is, the pressures below 150 MPa can affect the quaternary structure of proteins; 200 MPa can affect the tertiary structure, and 300–700 MPa can change the secondary structure [36,37]. Obviously, the pressure in our study is close to 10 MPa, which is not enough to change any structure other than the quaternary structure of the protein. Moreover, some related studies showed that high pressure does not affect the covalent bond of protein molecules, that is, it does not damage the primary structure of protein molecules–peptide bond structure [38]. Therefore, the second hypothesis is invalid, that is, under 103.4 bars, it is almost impossible for PLE extraction to destroy the polypeptide chain of the protein by pressure. Furthermore, combining the principle of the PLE, with a certain temperature, pressure can increase the permeability of the solvent, making it easier to enter the sample matrix and increase the contact time between the sample and the solvent [39]. Therefore, it can be inferred that the area change of different molecular weight bands is due to PLE changing the solubility of different molecular weight proteins in the extract, which leads to a higher content of small molecule proteins.

### 3.5. Polyphenol Profile of the Extracts: Triple TOF–LC–MS–MS

As polyphenols are active substances with strong antioxidant properties, it is valuable to analyze the specific phenol components in spirulina extracts. Therefore, in this study, PLE and non-pressurized extracts were analyzed by Triple TOF–LC–MS–MS. The effect of pressure treatment on the type of polyphenols was analyzed. 

**Figure 7:**
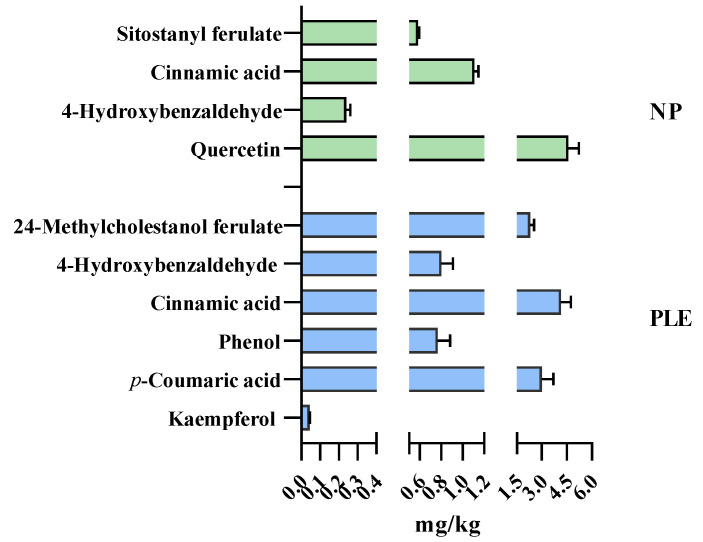
Concentration of phenolic components in PLE and non-pressurized extract.

Figure 7 shows the results of the type and content of polyphenol components. The results showed that different extraction processes can change the composition of polyphenols in algae extracts. For example, cinnamic acid and 4-hydroxybenzaldehyde were both detected in PLE and non-pressurized spirulina biomass. However, kaempferol, *p*-coumaric acid and 24-methylcholestanol ferulate were only found in PLE extracts while quercetin, and sitostanyl ferulate were found in the non-pressurized extracts. Moreover, PLE treatment increased the content of cinnamic acid (4.0 mg/kg vs. 1.0 mg/kg) and 4-hydroxybenzaldehyde (0.8 mg/kg vs. 0.2 mg/kg) when comparing with non-pressurized extracts. Moreover, the polyphenols in the PLE extract were mainly 24-methylcholestanol ferulate (2.3 mg/kg), cinnamic acid (4.0 mg/kg) and p-coumaric acid (3.0 mg/kg), while the polyphenols in the non-pressurized extracts were mainly quercetin (4.6 mg/kg). This shows that PLE can promote the recovery of a variety of polyphenols in Spirulina, which is an important reason for promoting the antioxidant capacity of the extract. Polyphenols in the biomass exist in free and bound forms, but in most cases, they are bound to other molecules such as proteins, structural carbohydrates, etc., by ester bonds through their carboxyl group or by ether bonds through their hydroxyl group [40]. In this study, it can be concluded that PLE changed the form of polyphenols in the extract. Considering the pressure treatment in the PLE extraction process, this is likely to promote the separation and release of bound polyphenols from spirulina.

## 4. Conclusions

The results obtained from this study showed that spirulina is a relevant source of nutrients and bioactive compounds with antioxidant capacity, but the recovery of these valuable constituents (proteins, polyphenols, carbohydrates and pigments) was greatly influenced by the extraction conditions (particularly temperature) explored in this study. Meanwhile, this study highlights the potential of PLE as an effective green extraction methodology to obtain high-added-value compounds from spirulina. Our study demonstrated that PLE may be a promising alternative for enhancing the selective extraction of antioxidant bioactive compounds from microalgae, which could be interesting for industrial upscaling. Finally, this study is the initial step for PLE to obtain natural active substances from spirulina; more applications of spirulina protein, carbohydrate and antioxidant substances in food, medicine and other industries need to be further developed.

## Figures and Tables

**Table 1:** Response surface methodology—central composite design.

Experiment	Temperature (°C)	Time (min)	pH
1	20	10	7
2	40	10	7
3	40	5	7
4	40	15	7
5	40	10	7
6	60	10	7
7	20	5	4
8	20	15	4
9	40	10	4
10	60	15	4
11	60	5	4
12	20	15	10
13	20	5	10
14	40	10	10
15	60	15	10
16	60	5	10

**Table 2:** Results of bioactive compounds and antioxidant capacity in spirulina extract.

Run	Protein %	Carbohydrate mg/g	Chlorophyll a mg/g	Chlorophyll b mg/g	Carotenoids mg/g	Total Polyphenol mg/g	ORAC µM TE
1	18.32 ± 1.10 ^abc^	12.98 ± 0.62 ^bcde^	0.267 ± 0.004 ^e^	0.524 ± 0.003 ^d^	0.092 ± 0.003 ^f^	1.59 ± 0.02 ^cd^	480.1 ± 5.5 ^c^
2	25.64 ± 0.00 ^de^	23.51 ± 0.75 ^f^	0.195 ± 0.003 ^e^	0.301 ± 0.001 ^c^	0.133 ± 0.004 ^g^	3.63 ± 0.08 ^fg^	365.9 ± 14.9 ^abc^
3	22.13 ± 0.00 ^cd^	26.15 ± 0.18 ^f^	0.283 ± 0.003 ^e^	0.527 ± 0.010 ^d^	0.137 ± 0.002 ^g^	4.64 ± 0.01 ^cde^	1491.0 ± 96.4 ^d^
4	29.82 ± 0.76 ^e^	39.83 ± 0.64 ^g^	0.436 ± 0.002 ^g^	0.600 ± 0.012 ^e^	0.265 ± 0.003 ^i^	6.23 ± 0.00 ^f^	1595.4 ± 96.4 ^d^
5	25.89 ± 0.72 ^de^	21.21 ± 0.65 ^f^	0.204 ± 0.010 ^e^	0.344 ± 0.002 ^c^	0.145 ± 0.000 ^g^	3.71 + ±0.08 ^f^	419.9 ± 58.9 ^bc^
6	19.48 ± 0.00 ^bc^	12.58 ± 0.57 ^bcd^	0.089 ± 0.001 ^b^	0.194 ± 0.007 ^b^	0.036 ± 0.002 ^d^	1.93 ± 0.01 ^b^	359.1 ± 47.8 ^abc^
7	15.21 ± 0.49 ^ab^	6.90 ± 0.25 ^a^	0.110 ± 0.000 ^c^	0.216 ± 0.002 ^b^	0.031 ± 0.000 ^cd^	0.97 ± 0.01 ^a^	148.1 ± 7.6 ^ab^
8	14.02 ± 1.08 ^a^	10.80 ± 0.37 ^abc^	0.057 ± 0.001 ^a^	0.120 ± 0.002 ^a^	0.004 ± 0.000 ^a^	0.63 ± 0.04 ^a^	129.2 ± 16.1 ^a^
9	47.87 ± 1.20 ^f^	80.95 ± 2.90 ^i^	1.406 ± 0.007 ^i^	2.017 ± 0.017 ^g^	0.648 ± 0.002 ^j^	11.61 ± 0.39 ^h^	2915.5 ± 371.4 ^e^
10	24.07 ± 1.60 ^d^	14.06 ± 1.06 ^cde^	0.061 ± 0.001 ^a^	0.106 ± 0.002 ^a^	0.013 ± 0.000 ^b^	2.79 ± 0.17 ^cde^	414.2 ± 48.3 ^bc^
11	15.15 ± 0.68 ^ab^	8.81 ± 0.14 ^ab^	0.058 ± 0.001 ^a^	0.129 ± 0.002 ^a^	0.015 ± 0.001 ^b^	1.61 ± 0.06 ^b^	247.0 ± 19.2 ^abc^
12	15.66 ± 0.51 ^ab^	15.55 ± 0.15 ^de^	0.225 ± 0.003 ^d^	0.487 ± 0.002 ^d^	0.085 ± 0.001 ^ef^	2.78 ± 0.07 ^de^	427.8 ± 30.6 ^c^
13	14.43 ± 0.00 ^a^	10.55 ± 0.38 ^abc^	0.090 ± 0.011 ^bc^	0.140 ± 0.003 ^a^	0.001 ± 0.000 ^a^	2.41 ± 0.02 ^g^	280.6 ± 34.7 ^abc^
14	45.81 ± 0.58 ^f^	58.90 ± 3.02 ^h^	0.516 ± 0.008 ^h^	1.626 ± 0.023 ^f^	0.002 ± 0.000 ^a^	10.85 ± 0.19 ^h^	2831.1 ± 295.5 ^e^
15	25.37 ± 1.21 ^de^	14.41 ± 0.40 ^cde^	0.058 ± 0.000 ^a^	0.095 ± 0.001 ^a^	0.029 ± 0.000 ^c^	2.75 ± 0.08 ^e^	468.5 ± 53.5 ^c^
16	19.33 ± 0.00 ^bc^	13.04 ± 0.36 ^bcde^	0.114 ± 0.001 ^c^	0.118 ± 0.001 ^a^	0.081 ± 0.001 ^e^	2.35 ± 0.06 ^c^	485.4 ± 61.6 ^c^

Note: the same letters in the same column of data indicate no significant difference (*p* > 0.05), and different letters indicate significant differences (*p* < 0.05).

## Data Availability

The datasets generated for this study are available on request to the corresponding author.
